# Hyperbaric oxygen therapy: More hope than hype for future treatment of perianal fistulizing Crohn's disease?

**DOI:** 10.1002/ueg2.12195

**Published:** 2022-01-10

**Authors:** Alessandro Armuzzi, Daniela Pugliese

**Affiliations:** ^1^ Dipartimento Universitario di Medicina e Chirurgia Traslazionale Università Cattolica del Sacro Cuore Rome Italy; ^2^ CEMAD IBD UNIT Unità Operativa Complessa di Medicina Interna e Gastroenterologia Dipartimento di Scienze Mediche e Chirurgiche Fondazione Policlinico Universitario “A. Gemelli” IRCCS Rome Italy

Perianal fistulas are a severe, disabling complication of Crohn's disease (CD) occurring in about 20% of patients within 20 years from diagnosis, with a significant impact on patients' quality of life and an increased risk of undergoing major abdominal surgery.^1^, ^2^ A multimodal approach, that is a combination of a surgical approach along medical therapies, mainly infliximab ± antibiotics, represents the current standard of care for treatment of complex perianal disease.^3^, ^4^ However, only a minority of patients achieve durable fistula remission, with high rate of recurrence, need for re‐intervention or diverting stoma.^5^, ^6^ The adjunctive topical treatment with expanded allogeneic adipose‐derived mesenchymal stem cells has demonstrated interesting results in the randomized double‐blind placebo‐controlled adipose derived mesenchymal stem cells for induction of remission (ADMIRE) trial, with up to 50% of fistula remission at week 24.^7^ However, the partial beneficial effect, the selective access criteria (no concomitant active luminal disease or active severe proctitis or diverting stoma) and the cost of each treatment denote that novel therapeutic strategies are increasingly needed for complex perianal CD.

Hyperbaric oxygen therapy (HBOT), involving intermittent inhalation of 100% oxygen at pressures >1 atm, has shown encouraging results in several CD phenotypes, including inflammatory luminal disease, enterocutaneous fistulas, metastatic perianal disease and fistulizing perianal disease.^8^, ^9^ The underlying involved mechanism seems to be related to the modulation of immune response and the promotion of tissue repair and wound healing mediated by hyperoxygenation.^10^

In the current study, Lansdorp et al. reported the long‐term (week 60) follow‐up of 20 patients enrolled in the HOT‐TOPIC trial.^11^ At enrolment, all patients had ≥1 actively draining high (defined as transversing the upper two‐thirds of the external sphincter/puborectal muscle, regardless of the number of internal and external openings) medical‐refractory perianal fistula for a median duration of 4 years (interquartile range 2–12 years). 16 patients (80%) were on concomitant biological therapy (14 on infliximab, one on vedolizumab and one on ustekinumab). Patients were treated with 40 daily sessions of HBOT on working days, for a total of 8 weeks.^12^ The co‐primary outcome was a comprehensive assessment of the fistula, with a clinical evaluation according to the perianal disease activity index (PDAI)^13^ and a magnetic resonance imaging (MRI) assessment through the modified Van Assche Index^14^ at week 16 and 60. At both time‐points, a significant reduction of the median PDAI score (8 vs. 4 vs. 4, *p* < 0.001 for both comparisons) and of the modified Van Assche Index (9.2 vs. 7.3 vs. 7.7, *p* = 0.004 and *p* = 0.005, respectively) was recorded compared to baseline. Moreover, at the end of follow‐up, 12 patients (60%) were in clinical remission (defined as a PDAI score ≤4) and four patients (20%) had a fibrotic fistula complex at MRI. Conversely, the improvement in biochemical (both C‐reactive protein and faecal calprotectin) and patient‐reported outcomes measures achieved at week 16 was no longer present at week 60. During the study, only three patients (15%) required perianal surgical re‐intervention. Overall, these results seem promising, especially considering the clinical features of the population enrolled. However, the greatest limit of this study is the absence of a control group to really assess the effect of the additive treatment with HBOT. As previously shown, in fact, in the ADMIRE trial where 34% of placebo‐treated patients achieved remission, an appropriate surgical procedure (fistula curettage, surgical drainage, and internal orifice closure) plus concomitant medications alone can significantly improve the fistula outcome. To be honest, a small control group of 8 patients unwilling to undergo HBOT was included, but no comparisons were possible.

Assuming a potential diffusion of HBOT in clinical practice for perianal fistula, three aspects should be considered: (1) the direct cost for health/insurance system; (2) the indirect cost for patients, treated for 40 working days and (3) the HBOT chambers availability across the IBD centres.

In conclusion, complex perianal fistulas represent one of the most challenging aspects of treatment of CD. Hyperbaric oxygen therapy seems to be a potential advance for standard‐of‐care refractory patients. Future larger studies are required to verify the real additive benefit and the cost‐effectiveness assessment of HBOT in this setting of difficult‐to‐treat patients.

## CONFICT OF INTEREST

The authors have no conflicts of interest to declare.
